# Climate warming accelerates somatic growth of an Arctic fish species in high-latitude lakes

**DOI:** 10.1038/s41598-023-43654-1

**Published:** 2023-10-05

**Authors:** Nicholas Kotowych, Aslak Smalås, Per-Arne Amundsen, Raul Primicerio

**Affiliations:** Faculty of Biosciences, Fisheries and Economics, UiT - The Arctic University of Norway, Tromsø, Norway

**Keywords:** Ecology, Climate-change ecology, Ecophysiology, Freshwater ecology, Population dynamics

## Abstract

High-latitude aquatic ecosystems are responding to rapid climate warming. A longer ice-free season with higher water temperatures may accelerate somatic growth in lake ectotherms, leading to widespread ecological implications. In fish, rising temperatures are expected to boost rates of food intake and conversion, and predictions based on empirical relationships between temperature and growth suggest a substantial increase in fish growth rates during the last decades. Fish abundance negatively affects growth by limiting food availability. This field study addresses the effects of climate warming on growth of a subarctic population of Arctic charr (*Salvelinus alpinus* (L.) over nearly 40 years. Juvenile growth of 680 individuals of Arctic charr, was reconstructed by sclerochronological analysis using sagittal otoliths sampled annually from the early 1980s to 2016. Statistical modelling revealed a positive effect of water temperature, and a negative effect of abundance on somatic growth in juvenile individuals. Temperature dependence in growth was significant for average and fast-growing individuals across all investigated age classes. These findings suggest that, as temperatures rise, somatic growth of Arctic charr will increase in high latitude lakes. Climate warming will thus influence cold water fish life history and size-structured interactions, with important consequences for their populations and ecosystems.

## Introduction

Water temperature is conceivably the most important eco-physiological factor affecting the performance of aquatic ectotherms^1,2^. Biological processes, rates and functions such as metabolic rate, locomotion, foraging ability, and rates of feeding and growth are all influenced by temperature^1,3^. Temperature dependent growth plays a prominent role in mediating the ecological effects of climate change as it indirectly affects life history, demography and size-structured interactions^4^. In fish, the effects of climate warming on growth warrant closer scrutiny as they indirectly influence survival, maturation and reproductive success^5–7^. Cold-water fish species living in high latitude lakes are described among the most sensitive to climate warming^8,9^, and are experiencing faster temperature increases than fish living at lower latitudes^10^. However, there is presently scarce empirical evidence of climate driven change in fish growth rates at high latitudes due to a lack of long-term studies and the possible masking effects of population abundance^11^.

Fish growth rates increase with temperature, up to an optima, beyond which they decline, suggested to be caused by metabolic costs exceeding energy intake^5,6,12^. The empirical relationship between water temperature and growth rate differs greatly between fish species^7^. Accordingly, fluctuations and increase in water temperature due to climate variability and warming will have effects on fish growth that are species specific^13,14^. Cold-water adapted fish species are expected to respond promptly to global warming due to their sensitivity to increasing water temperatures and to the higher rate of change observed at high latitudes^15^. In the northern reaches of their distribution, salmonids and other cold-water adapted species experience temperatures in the lower end of their tolerance range, and therefore warming could improve their growth performance^4^.

In addition to water temperature, fish somatic growth rates are strongly influenced by food supply and there is a close relationship between ingestion rate and growth rate^16^. Further, ingestion rate and food availability plays a pivotal role in mediating temperature-dependent growth effects, for instance shifting optimal temperatures for growth based on the availability of food^17^. When ingestion rates are low, the highest growth rate occurs at low temperatures, but with increasing ingestion rates the optimal temperature for growth tends to increase^16,17^. Consequently, individual fish growth is contingent and directly related to food acquisition rate^16,18^. Prey availability, and hence food supply, for fish is negatively influenced by the abundance of competitors, and higher abundances reduce food intake resulting in lower growth rates^19,20^. Moreover, climate warming may exacerbate density-dependence in lakes, by promoting increased population abundance and competitive pressure on foraging opportunities^21^.

Arctic charr (*Salvelinus alpinus* (L.) is the most cold-water adapted salmonid^22^, capable of surviving and feeding at temperatures as low as ~ 0 °C^13^. Conversely, Arctic charr are the least tolerant of high-water temperatures, with critical thermal limits for survival at 26–27 °C, compared to 30–33 °C for Atlantic salmon and 26–30 °C for Brown trout^2^. For most populations of Arctic charr, growth may begin at 1–3 °C^14^**,** and the temperature range within which they may feed while exhibiting no signs of abnormal behavior is 3–16 °C^23^. The effect of temperature on the growth of Arctic charr has been studied extensively. Laboratory experiments have demonstrated that increasing temperatures are associated with increased growth, up to an optimum range of 11–14 °C past which growth steeply declines^5,6,14,23^. Given the temperature affinity and sensitivity of Arctic charr, it is difficult to obtain field evidence of positive climatic effects on wild charr growth due to the paucity of long-term ecological studies at high latitudes^24,25^.

In this study we provide unique field evidence of the effects of climate change on wild Arctic charr growth over nearly 40 years of warming. The study system is well suited to explore growth effects because of the long time-series that include a large variation in both experienced water temperatures and in the density of competitors caused by a culling experiment (see material and methods). Climate driven water temperature change at these high latitudes should increase somatic growth for cold-water adapted species, an expectation that has not been addressed in past climate change impact research. Emphasis is placed on the juveniles when the effect of temperature on growth is particularly evident^7,26^. Temporal variation in growth over four decades (1986–2016) is assessed based on length-at-age estimates obtained from Arctic charr individual measurements and otolith readings^27^. Age specific length increments are then modelled to investigate the effect of water temperature on somatic growth having accounted for negative density-dependence. Supporting evidence for the positive effect of warming on somatic growth of cold-water adapted species would imply that eventual climatic risk is not direct, but contingent on interactions with species adapted to warmer water.

## Materials and methods

### Study location

The Arctic charr individuals (N = 680) were sampled from Takvatn, a subarctic lake located in Northern Norway (69° 07′ N, 19° 05′ E) from the year 1986 to the year 2016. The lake is oligotrophic, dimictic, and situated 214 m above sea level. Takvatn has a surface area of 14.2 km^2^ with an approximate depth of 80 m^28^. The climate is subarctic, with an average air temperature in July of 13.2 °C. The lake is ice free from end of May or early June to November^28,29^. Mid-summer surface water temperatures are approximately 12 °C, with a maximum temperature of 14 °C^29^, followed by autumn circulation which brings winter temperatures to 2 °C or lower^28^. Takvatn supports a fish community composed of Arctic charr (*Salvelinus alpinus*, hereafter charr), Brown trout (*Salmo trutta*, hereafter trout), and three-spined stickleback (*Gasterosteus aculeatus*), which are all permanent lake residents^28^.

### Field sampling

Over the study period (1986–2016), field sampling procedures were standardized and fish processing was conducted similarly. Monofilament gill nets were set in the month of August in the littoral zone (< 15 m) of the lake for approximately the same number of fishing hours (12 h) and the same multiple sampling sites across all sampling years. Multi-mesh gillnets were used (Norwegian ‘Benthic Gillnet Overview’ (BGO) gillnets (L = 40 m, W = 1.5 m)) to catch a representative sample of all size-classes of the fish present in the lake. BGO gillnets have 10, 12.5, 15, 18, 22, 26, 35 and 45 mm mesh sizes, measured from knot to knot, and alternate at 5 m intervals horizontally. This sampling procedure was conducted to collect data both on individual fish for otolith measurements and for catch-per-unit-effort (relative abundance) estimates. For additional information and a detailed description of Takvatn and field sampling procedures refer to Klemetsen et al.^30^.

### Temperature

Water-temperature measurements in Takvatn were not sufficiently frequent to provide a quantitative assessment of the variation in thermal environment experienced during the study. Therefore, we used historical data from the nearby meteorological station at Bardufoss Airport (~ 19 km from Takvatn), to force a one-dimensional air-to-water temperature model, the General Lake Model available in R (v.3.6.0)^31^ with package GLMr^32^. This temperature model assumes no horizontal temperature variability within the water body and computes vertical temperature profiles by accounting for surface heating, surface cooling, and vertical mixing. The model also includes the effects of ice-cover formation and subsequent melting, heating and mixing processes within the lake^32^. We calibrated the model using water-temperature data from Takvatn for the years 1982, 1992, 1994, 1997, and 2017. The air-to-water temperature model requires input data for climatic variables (air temperature, precipitation, solar radiation, wind speed, cloud cover, and relative humidity) and lake-morphometric variables^32^. To evaluate model performance, we used daily measured water temperature data from 2018 to 2019 (Fig. B1, Supplementary information). The air-to-water temperature model performed reasonably well, with little discrepancy between modelled and observed water temperature, thermocline depth and Schmidt stability in the evaluation years (2018 and 2019) (Fig. B2, Supplementary information).

### Catch per unit effort data

Catch per unit effort (CPUE) data were collected by gillnetting fish in the littoral zone (< 15 m) of Takvatn. The CPUE data have the unit of number of fish individuals caught per 100 m^2^ per net night, the CPUE data was estimated for catches only in the month of August each year (the month where fishing effort was standardised each year). Refer to^30^ for a detailed overview of Takvatn CPUE and the culling which removed 666 000 individual charr from the lake between 1984 and 1989. The culling was conducted as an experiment to change the dynamics and the stable state in the charr population^33^, from slow-growing, highly parasitized old individuals, to a younger population with less parasites and faster somatic growth rate more valuable for human consumption^30^. The relative abundance (CPUE) of charr declined considerably during the late 1980s. Prior to the culling, the lake inhabited a high density of small and slow-growing charr (CPUE = 17.5 fish/100 m^2^ gill-net/night) and very few trout (CPUE = 0.1 fish/100 m^2^ gill-net/night)^30^. Eventually, after the culling, the CPUE of both species stabilized to similar levels^27^.

### Otoliths

Sagittal otoliths (i.e. ear bones or stones) extracted from charr at the Takvatn field station were analyzed in the laboratory to determine age and back-calculate size at age of individual fish. The total number of fish sampled for age determination and growth estimation purposes was 680. The otoliths analyzed were distributed over 33 years encompassing extensive climatic variability, with marked inter-annual differences in mean air temperatures. Otolith measurements and aging were validated by randomly selecting 40 otoliths, which were independently measured and aged a second time by two researchers, to evaluate measurement precision and age determination consistency. Glycerol was used to increase clarity of annual growth zones^34^ and facilitate handling. Each otolith was positioned with the posterior nucleus side facing upwards and the sulcus-side downwards. A stereomicroscope and integrated camera (Carl Zeiss SteREO Discovery. V8; Zeiss Axiocam ERc5s) were used to photograph otoliths, with components set as follows: objective, 1; optovar, 3.2x. One picture was taken of each otolith, and two copies were made, one a raw image, which did not include any measurements and was kept as a control for validating age and growth measurements, and a second image, used to measure annual growth zones in a separate file. All images were analyzed with Carl Zeiss—Zen (blue edition) 2011, software (version 1.0.0.0).

To assess the age of each otolith, hyaline and opaque zones were counted until the last full year of growth. Once the fish was aged, growth was evaluated by measuring the width of annual growth increments. The first measurement taken was otolith radius, a transect was initially positioned from the center of the primordium to the last full year of growth, on the ventral side, toward the anterior end of the otolith; the resultant total otolith length is representative of total fish length. Next, another transect line was positioned from the centre of the primordium, at the same location as the first transect above, and stretched out to the end of the nucleus, the resultant transects produced a length measurement for the first year of growth. Following these two transects, additional measurements were made by positioning transects at the beginning of each subsequent hyaline zone to the end of the proceeding opaque zone, resulting in annual length measurements, and this was done for each full year of growth (both hyaline and opaque zone). For more details on otolith treatment see Kotowych^35^.

### Statistical analysis

Temporal trends in mean annual autumn water temperature (MAT) from 1983 to 2016 were studied using linear regression. In slow growing species such as Arctic charr^36^, back-calculation of length at age may be impaired by the uncoupling between somatic and otolith growth. This is known as an age-effect whereby as the fish gets older and somatic growth stagnates otolith accretions may still develop^37,38^. To address the age effect present in charr otoliths^36^, the statistical model for back-calculation proposed by Finstad^39^ was applied. The model adds an interaction term to a back-calculation method developed by Morita & Matsuishi^36^, where an age effect is included to account for the otolith size increasing continuously during periods with little, or no somatic growth (i.e. uncoupling between somatic and otolith growth)^39^:

$${\text{L}}_{{\text{t}}} = \left[ {{\text{O}}_{{\text{t}}} {\text{O}}_{{\text{T}}}^{{ - {1}}} \left( {\beta_{0} + \beta_{{1}} {\text{L}}_{{\text{T}}} + \beta_{{2}} {\text{T}} + \beta_{{3}} {\text{L}}_{{\text{T}}} {\text{T}}} \right) - \beta_{0} - \beta_{{2}} {\text{t}}} \right]\left( {\beta_{{1}} + \beta_{{3}} {\text{t}}} \right)^{{ - {1}}}$$

where L_t_ is the back-calculated length at age t, T is age at capture, O_t_ is the measured otolith radius at age t, O_T_ is the observed otolith size at the time of capture and L_T_ is the observed fish length at the time of capture. The β_0,_ β_1_, β_2_ and β_3_ are coefficients estimated by least squares multiple regression^39^. An evaluation of back-calculated lengths showed a good performance of the Finstad^39^ model, with a satisfactory agreement between back-calculated lengths (i.e. sum of all back-calculated yearly length increments for each fish) and observed lengths at capture (R^2^ = 0.93; *p* < 0.001). The available data for Arctic charr allowed for back-calculation of lengths for age classes 1 to 4 years, as older age classes had too low sample size for reliable statistical analysis. Parametric regression models were used to investigate the effects of inter-annual variability in water temperature and relative abundance (CPUE) on somatic growth across age classes of Arctic charr. Linear regression models were used to study temporal trends in length increments of juvenile charr (aged 1–4 years) over the study period (1986–2016).

To study abundance and temperature effects on average growth, Linear Mixed-Effect models (LME) were applied. The response variable is therefore back-calculated yearly length increments of 1–4-year-old charr individuals which is a continuous variable. To not violate model assumptions of normal distribution in the residuals we log transformed the response variable. Model diagnostics plots are included in the supplementary information (Fig. A1, supplementary information), which show that the assumption of normal distribution of the residuals are met and that there is no heteroscedasticity in the residuals and no outliers that have high influence on the model fit. We defined each fish with a random intercept to account for the repeated measures in the otolith data used here. In addition, we defined year with a random intercept to account for between-year differences not explored in our model. To answer the hypothesis of how climate warming and temperature variability affects juvenile growth in Arctic charr we included water temperature in the same year as the growth estimates as a continuous fixed explanatory variable. We chose mean water temperature of the upper ten meters of the water column as this is the depth most utilized by Arctic charr^40^. Several studies from Lake Takvatn describes the habitat use of Arctic charr, where the vast majority of fish uses the shallow water habitat (> 10 m of depth)^41^, in addition, charr caught in the littoral zone show a high degree of stationarity^42^. Furthermore, we opted to use the mean water temperature from September through November (MAT) which informs of the temperature experienced in late summer, but more importantly also a description of the duration of the growth season. Variation in autumn temperature and timing of ice formation was greater than the variation in summer water temperature and timing of ice breakup (Fig. B3, supplementary information). This means that the potential of between-year variation in somatic growth of charr is larger during autumn and thus autumn temperature is a more suitable candidate explaining difference in somatic growth caused by temperature. In addition, mean autumn temperature was the water temperature variable (also tested summer (jun-aug) and annual mean water temperature) explaining most of the variation in juvenile (age 1–4 years) back-calculated growth of Arctic charr according to multiple model selection criteria (AIC, BIC and log-likelihood) (Table A2, supplementary information). Age of the charr, from 1 to 4 years old, was added as a fixed categorical explanatory variable as it was expected that somatic growth varies greatly with age because of their potential dietary shifts within the upper water column as they become larger (older)^43^.

To study how population density affects juvenile growth of the charr, we included a measure of relative density (CPUE), which is a continuous fixed explanatory variable. Furthermore, we separated the estimated relative density (CPUE) of Arctic charr and Brown trout based on a size-threshold for which individuals that potentially would be competitors (CPUEc) and which would be predators (CPUEp) for the juvenile Arctic charr (age 1–4). Arctic charr below 30 cm and trout below 15 cm were classified as competitors for the juvenile Arctic charr individuals in this study (see^43^ Prati et al. 2021, for description of the diet of the different size-classes). The potential predators for these juvenile Arctic charr were defined as trout above 25 cm^43^. Both CPUEc and CPUEp were included in the mixed effect model to investigate how relative density of both competitors and predators affected juvenile somatic growth of Arctic charr.

We then used model selection by the Bayesian Information Criterion (BIC), which is a conservative model selection criterion penalizing model complexity more than other model selection methods. All possible combinations of fixed variables were allowed to interact in addition to being additive (see Table A1 for model selection results). The final model structure was,

$$Y_{ijk} = a_{k} + a_{Yk} + a_{Yi} + \beta_{Aj} + \tilde{X}_{k} + \tilde{Z}_{k} + C_{{\beta_{Aj} \tilde{X}_{k} }} + \varepsilon_{ijk} N\left( {0, \sigma^{2} } \right),$$

where ${y}_{ijk}$ is the annual length increment (mm) for the $i$ th fish at age $j$ in year $k$, ${a}_{k}$ is the fixed annual growth increment, ${a}_{Yk}$ is the random intrinsic fish effect, ${a}_{Yi}$ is the random intrinsic year effect,${\beta }_{Aj}$ describes the linear age-dependent change in growth, ${\widetilde{X}}_{k}$ and ${\widetilde{Z}}_{k}$ is the autumn water temperature (MAT) and relative density of competitors (CPUEc) in year $k$, respectively, ${C}_{{\beta }_{Aj}{\widetilde{X}}_{k}}$ is the interaction effect between the age-dependent growth and the mean autumn temperature at year k, and $\varepsilon$ is assumed to be independent errors with mean zero and a common variance ${\sigma }^{2}$. Continuous explanatory variables were centred and standardized to compare their effects (MAT, mean = 6.18, SD = 0.714, CPUEc, mean = 9.82, SD = 4.15). The effect of potential predators (CPUEp) was therefore dropped in the final model, together with other interaction effects not described in the model above (Table A1, supplementary information). We assessed the potential presence of autocorrelation in the input variables by examining autocorrelation plots generated using the acf function from the tseries package in R. Our analysis revealed no indications of autocorrelation in the input variables (Fig. A2, supplementary information). Furthermore, we conducted the Durbin-Watson test to evaluate autocorrelation in the residuals of the model. The test statistic for our model was 1.63, indicating the absence of autocorrelation issues with a test statistic value close to 2^44^. To assess multicollinearity among the model variables, we utilized the vif function (Variance Inflation Factor) from the car package in R. The results indicated either no or minimal collinearity as all values are between 1.14 and 1.3, which is well within the acceptable level of collinearity^45^.

To investigate the influence of density and temperature on charr with growth performance above or below the average (average was explored in the mixed model), we employed quantile regression models. Specifically, we focused on the 50th, 75th, and 90th percentiles of growth as separate quantiles of interest. By analyzing these quantiles, we aimed to capture potential variations in the relationship between abundance, temperature, and growth among individuals displaying exceptional growth rates compared to the average population, providing a more nuanced understanding of their interactions. Computations, statistical analyses and graphics were made in R (version 3.6.1^31^ using the packages “lme4”^46^ for Linear Mixed-Effect models, “quantreg”^47^ for quantile regression model estimation, “sjPlot”^48^ for tables production, and “ggplot2”^49^ for plotting.

## Results

**Figure 1:**
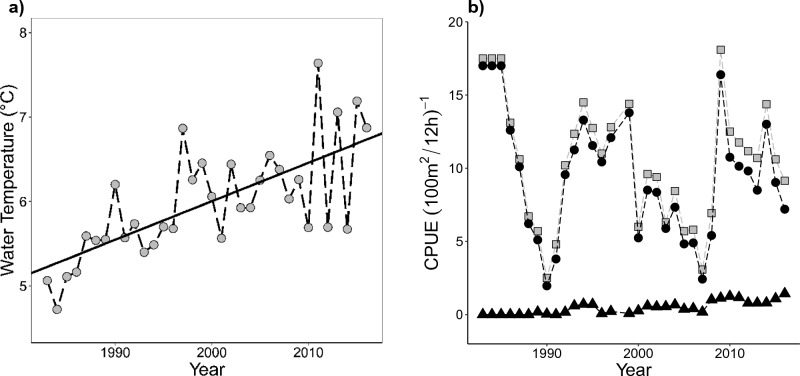
(**a**) Modelled mean autumn (September–November) water-temperature, with corresponding linear regression trend line, and (**b**) the combined relative density of charr and trout (CPUEtot, grey squares), relative density of competitors (Arctic charr < 30 cm and brown trout < 15 cm) (CPUEc, black dots) and relative density of potential predators (brown trout > 25 cm) (CPUEp, black triangles) in Lake Takvatn from 1983 to 2016.

The autumn water-temperature increased significantly throughout the study period (year 1986–2016) by 0.46 ˚C per decade (F_1, 32_ = 30.48, R^2^ = 0.472, *p *value < 0.001), starting at around 5.18 ˚C in the 1980s and increasing to about 6.75 ˚C by the end of the study period (Fig. 1A). The combined relative density (CPUEtot) for charr and trout, which showed a decline in the 80 s in concomitance with culling, did not display a significant temporal linear trend (F_1, 31_ = 0.84, R^2^ = 0.02, *p* value = 0.37) during the study period (Fig. 1B). The relative density of potential competitors (CPUEc, charr < 30 cm and trout < 15 cm) for juvenile charr (aged 1–4) follows closely the CPUEtot and shows no linear trend over the study period (F_1, 31_ = 2.06, R^2^ = 0.06, *p* value = 0.16). However, the development in potential predators across the study period shows a slight increase from the 1980s to 2016 (F_1, 31_ = 64.71, R^2^ = 0.66, *p* value < 0.001) (Fig. 1B).

**Figure 2:**
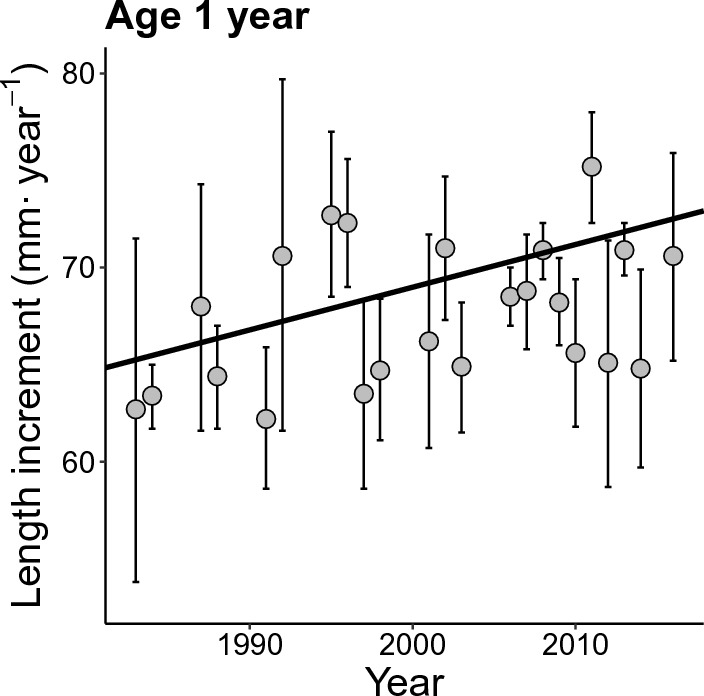
Estimated per-year average length increment (mm·year^−1^) for 1-year-old Arctic charr in Lake Takvatn from 1983 to 2016. The trend line was estimated by linear regression. Error bars show the 95% confidence interval for mean length increment.

Fish growth showed a significant increase over the study period (Fig. 2). Yearly length increment of 1-year-old charr increased by nearly 10% from the year 1983 to 2016, from 64 to 70.5 mm (F_1, 678_ = 35.18, R^2^ = 0.0479, *p* value < 0.001). A significant, positive trend in somatic growth was found in all age classes included in the study (Supplementary information, Table C1–C4, Fig. C1).

**Figure 3:**
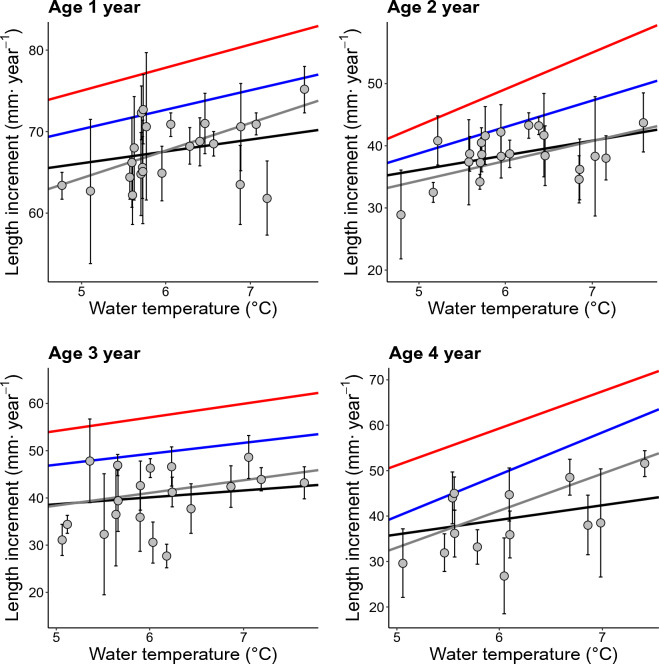
Estimated per-year average length increment (mm·year^−1^) plotted against autumn mean water temperature (MAT) (°C) by age group. The plots for the four age groups include linear and quantile regression lines, with mean, being estimates from the global mixed effect model (black line), median (grey line), 75th (blue line) and 90th (red line) percentiles. Error bars show the 95% confidence interval for mean length increment.

Mean autumn water-temperature was significantly and positively related to average growth in the linear mixed-effect model for all age classes (Fig. 3). The median and fast growing (75th and 90th percentiles) charr showed similar relationships with temperature as seen for the whole population (Fig. 3). According to the linear mixed-effect model, mean annual autumn water temperature affected length increment in the various age groups differently. Length increment for the 1-year-old age class showed a positive relationship with water temperature, displaying a 6.8% (4.18 mm) increase in length increment over the experienced temperature range in this study (4.72–7.63 °C) (LME-model, Conditional R^2^ = 0.57, *p* < 0.001) (Fig. 3, top left panel, supplementary information Table A3). The median and fast growing 1-year-olds (quantile regression) show similar, but somewhat larger increase in length increment with temperature (Fig. 3, top left panel, supplementary information Table A4). Length increment for the 2-year-old age class also showed a positive, but somewhat steeper, relationship with temperature, with a 12.4% (4.4 mm) increase in somatic growth over the experienced water temperature range (4.72–7.63 °C), with median and fast-growing fish displaying similar trends (Fig. 3, top right panel. Supplementary information, Table A5, quantile regression).

The effect of water temperature on length increment for the 3-year-old age class was weaker, and not significantly different from the 1-year-old age class (*p* = 0.056), with a relative increase of 11.1% (4.18 mm) in length increment over the experienced range of water temperature (4.72–7.63 °C) (Supplementary information for the LME-model results Table A3). Similar trends were true for the median and fast-growing individuals (Fig. 3, bottom left panel and supplementary information Table A6). The strongest effect of water temperature was on the 4-year-old age class with an increase of 12.6% (4.52 mm) in length increment over the experienced range in water temperature (4.72–7.63 °C), with a steeper trend for median and fast growing charr individuals (Fig. 3, bottom right panel and supplementary information Table A3, and Table A7 for quantile regression model results).

**Figure 4:**
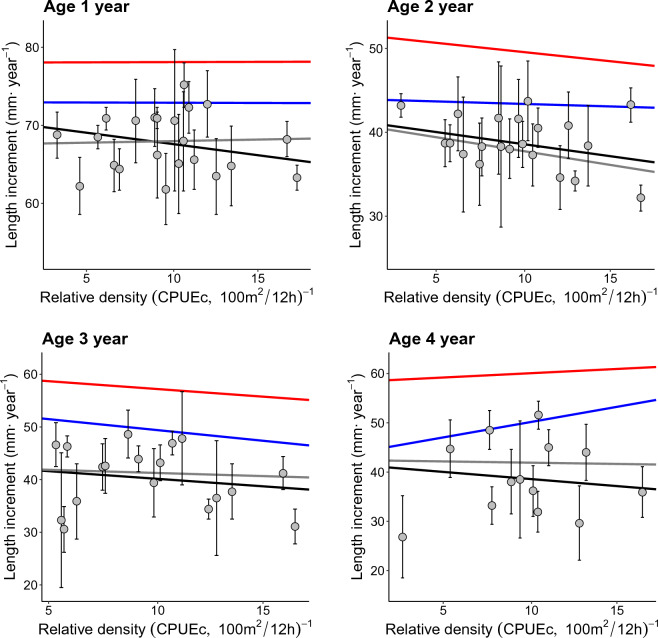
Estimated per-year average length increment (mm year^−1^) plotted against relative density of competitors (Arctic charr < 30 cm and brown trout < 15 cm) (Catch-Per-Unit-Effort-competitors, CPUEc), given as number of fish 100 m^2^ net-night^−1^, by age group. The plots for the four age groups include linear and percentile regression lines, with mean (black line), median (grey line), 75th (blue line) and 90th (red line) percentiles. Error bars show the 95% confidence interval for mean length increment.

Increased relative density of competitors (CPUEc, charr < 25 cm and trout < 15 cm) accounted for some decrease in somatic growth, although the effect did not significantly differ with the age of charr individuals. Additionally, the effect was different depending on whether the mean (black line, from the LME-model), median (grey line), 75th percentile (blue line) or 90th percentile (red line) of the population were considered (Fig. 4). Length increment decreased with 3.6 mm over the experienced range of relative density of competitors (CPUEc), which translated to 5.7, 10.1, 9.7, and 9.9% decrease in relative length increment for age 1–4-year-old, respectively (LME-model, Conditional R^2^ = 0.57, *p* = 0.047) (Supplementary information Table A3). For the median and fast growing individuals the results were variable between age groups, chiefly showing a negative relationship between somatic growth and relative density of competitors (CPUEc), but not for all age classes and not consistently for fish with faster than average growth (Fig. 4, and supplementary information Table A4–A7).

Autumn water temperature had a stronger effect than relative density on temporal variation in individual fish growth for all age classes in this study (Supplementary information, Table A3). For example, for the 4-year-old age group of charr, growth increased by 1.11 mm per standard deviation of increase in water temperature (LME-model, Conditional R^2^ = 0.57, *p* < 0.001), but decreased by 0.9 mm per standard deviation of increase in CPUEc (LME-model, Conditional R^2^ = 0.445, *p* = 0.047) (Supplementary information, Table A3).

## Discussion

Our findings demonstrate that field studies can effectively examine the impact of climate change on fish populations by employing a long-term, multidecadal approach. Significantly, we provide the first empirical evidence of climate change directly influencing fish growth at high latitudes, where temperatures are rapidly increasing. Specifically, we observed a substantial increase in the growth rates of juvenile Arctic charr with warming, along with a notable negative impact of population density. The positive relationship between temperature and growth was evident across all age classes of the charr population, which is a cold-water species with the lowest temperature optimum and affinity. The gradual warming trend observed in the lake over the study period (1983–2016) coincided with a corresponding positive trend in juvenile charr growth, strongly suggesting that climate change has already influenced Arctic charr at these latitudes. Importantly, we also identified a significant negative impact of relative abundance on somatic growth in juvenile charr (ages 1–4), emphasizing the importance of accounting for density-dependence in field studies investigating the effects of climate change on fish life history. This finding implies that indirect effects of climate change mediated by alterations in prey numbers, or the density of competing species may pose a threat to wild populations of cold-water species under warming.

The four decades of growth data under climate warming provide compelling evidence of temperature-dependent growth in wild Arctic charr, which is consistent with experimental laboratory findings^6^. Previous field studies have produced inconclusive results regarding the effects of temperature on growth, likely due to limited temperature variability in short-term studies and significant within-year variation in individual growth that masks population-level effects^24,25,50^. However, it has been previously demonstrated that the body size of young-of-the-year fish increases with water and air temperatures at high latitudes^50^. Over the study period, we observed an approximately 10% increase in length-at-age for 1-year-old juvenile charr, making them nearly a centimeter larger than individuals caught 40 years ago. Young-of-the-year fish from many species tend to prefer warmer waters compared to larger conspecifics, possibly to maximize growth and increase their chances of successful over-wintering^7,22,28,50,51^. The influence of temperature on growth varies depending on the age, ontogenetic stage, and size of the fish^5,7^. Interestingly, 4-year-old charr displayed the most pronounced increase in growth with temperature. These individuals may choose the more profitable littoral zone to a greater extent than their younger conspecifics, as the risks associated with predation decrease and competitive strength among salmonids increases with larger body size^28,52^. Additionally, 4-year-old individuals have a wider dietary niche and access to more nutritious food due to their larger size, enhancing their ability to meet metabolic demands^43^. Moreover, our analysis revealed a significant inter-individual variation in growth across all age groups, indicating substantial differences in temperature selection and food acquisition rates among individuals. This variation underscores the complex nature of fish growth responses to environmental factors, which highlights the importance of considering individual-level variations when studying the effects of i.e. temperature on fish growth.

In light of the significant inter-individual variation in Arctic charr growth we showed by using quantile regression that individuals with median to fast somatic growth were more responsive to higher water temperatures compared to slower-growing charr. This within-year variation in growth among individuals can be partly attributed to the distinct thermal regimes experienced in different habitats commonly utilized by salmonids, such as the littoral, pelagic, and profundal zones of deeper lakes like Takvatn^22,28^. The adaptive utilization of these habitats is influenced by factors such as food profitability, competition, and predation risk^28,51,53,54^. In the warmer littoral zone, charr individuals would encounter the most pronounced warming trends, but compensate for increased metabolic demands through enhanced foraging opportunities, thereby resulting in higher growth rates^12,24,55^. In contrast, individuals inhabiting the cold profundal zone would experience only a moderate temperature increase in a less productive habitat, leading to minimal or negligible changes in growth^30,51^. Another potential explanation is that fast-growing individuals may possess superior competitive abilities, allowing them to outcompete their conspecifics and achieve higher somatic growth rates^56^. This is supported by the findings of the quantile regression analyses, which demonstrated that the fast-growing individuals did not exhibit the same consistently negative relationship in growth with increasing relative density as the rest of the juvenile population.

An increase in the relative density of charr (< 25 cm) and trout (< 15 cm) had a significant negative effect on the growth rate of juvenile Arctic charr in Takvatn. Previous studies have demonstrated that high charr abundance can have considerable adverse impacts on food availability, somatic growth, and population dynamics in salmonids^18,19,27,28,30^. For example, before the culling experiment in the mid-1980s, Arctic charr in Takvatn exhibited a unimodal size distribution and smaller overall body sizes, which were associated with reduced food consumption^19,30^. The availability of food in Takvatn is closely linked to fish abundance and competition, which likely played a crucial role in the inter- and intra-annual variation in the somatic growth rate of charr^28,29,57^. While the impact of trout on charr growth and habitat use has been documented in several lakes^58^, it was considered less influential than intraspecific competition in Takvatn in previous studies that lacked information on individual charr growth^18,33^. However, given that trout is a stronger competitor than charr in warmer water temperatures, it may become a more significant competitor for charr with further climate warming^58^.

The relationship between relative density and juvenile somatic growth was weaker than that of water temperature in this study, suggesting that the experienced density range permitted an increase in growth enabled by the increase in water temperature. In fish, the availability and quality of food plays a crucial role in enabling the growth benefits of increased temperatures^24,25^. In ectotherms, higher temperatures increase metabolic demands, and if not met with increases in food consumption, may result in adverse effects^24,55^. In charr, the optimum temperature for growth of 14.1 °C derived from laboratory studies is for fish being fed on maximum rations; this optimum varies strongly with ration sizes^2,6^. In addition, food availability is also impacted by lake productivity, with Lake Takvatn and other lakes at these latitudes being typically unproductive and oligotrophic^27^. However, climate warming is projected to increase productivity in these lakes by increasing nutrient loads^59^. An increase in productivity might accentuate the effects of higher water temperatures on somatic growth by increasing food availability for salmonids living in these lakes. Productivity changes as a result of climate change was not studied here but could also have contributed to the observed changes in somatic growth of juvenile charr.

Several other factors not studied here could influence growth in fish, for instance lag effects from early life stages, change in water quality, genetic factors, and environmental disturbances. Lag effects from early life stages, such as embryonic development and larval conditions, can have long-term impacts on growth trajectories^60^. Changes in water quality parameters, such as oxygen levels and pH, can affect metabolic efficiency and physiological performance, potentially impacting growth rates^61^. Genetic factors, including growth-related genes and heritable traits, contribute to growth variation among individuals within charr populations^62^. The charr population in Lake Takvatn have little genetic variability as it was introduced, but only very few individuals, from a nearby lake in the 1950ies (see^30^), making growth differences less likely to be explained by genetic differentiation between individuals. However, all these factors need to be considered in the context of climate change, as changing environmental conditions may amplify their effects and pose additional challenges for the growth and survival of Arctic charr populations.

The effects of climate warming on growth documented in this study have several implications for fish in comparable systems across the Holarctic. If temperatures increase as anticipated, cold-water fish species will correspondingly increase in size, granted that thermal tolerances are not exceeded, and food availability meets the higher metabolic demands. In sub-Arctic areas, water temperatures are projected to increase substantially towards the year 2100, however temperatures are unlikely to exceed Arctic charr’s (~ 14 °C) and brown trout’s (~ 16 °C) optimum for somatic growth for the majority of the growth season^4^. More warm water adapted species like trout will have greater growth benefits from warming than colder water adapted species like charr^63^, possibly leading to a competitive advantage for trout^58^. Predictive modelling studies have already shown that size at age for cold water salmonids living at high latitudes will likely increase as climate warms^4,21^. Climate driven growth acceleration has widespread ecological implications, given that body size influences maturation schedules and fecundity, competitive ability, strength of predator–prey interactions, and energy fluxes in food webs^64^. Further, through its influence on growth, climate warming is expected to interact with size-selective harvesting by angling and gill nets, which will target younger fish, including immatures, accentuating age-truncation and eventually reducing recruitment, resulting in increased vulnerability of fish populations to further environmental perturbations^4^.

## Conclusions

At the high latitudes of subarctic Lake Takvatn, climate warming has been rapid during the last four decades leading to a considerable increase in growth of Arctic charr, the species with the lowest temperature affinity. Currently, young charr individuals grow on average by one additional centimeter each year relative to their conspecifics 40 years ago. The clear growth benefits of warming in Arctic charr indicate that the direct effects of temperature increase will not be a threat to cold water species in subarctic regions. The documented strong negative density-dependence in growth rather suggests that climate change risk will emerge from indirect effects mediated by changes in resource availability and numbers of competitors. This study also shows the importance of using a long-term, multidecadal approach for detecting the relatively small and gradual changes caused by climate warming. By using this approach, we have for the first time confirmed empirically in wild populations the predictions from laboratory and simulation studies.

### Supplementary Information

Supplementary Information.

## Data Availability

Datasets used for the analysis will be made available in a Dryad-repository upon acceptance of the MS, but will also be available through reasonable request made to the corresponding author.
