# Supplementary material for: Climate warming accelerates somatic growth of an Arctic fish species in high-latitude lakes
Source: Sci Rep. 2023 Oct 5;13:16749. doi: 10.1038/s41598-023-43654-1 (PMC10556027; doi:10.1038/s41598-023-43654-1)
Supplement: Supplementary file 1 — Supplementary Information. [file 41598_2023_43654_MOESM1_ESM.docx]

**Climate warming accelerates somatic growth of an Arctic fish species in high-latitude lakes**

Nicholas Kotowych, Aslak Smalås*, Per-Arne Amundsen, Raul Primicerio

UiT - The Arctic University of Norway, Faculty of Biosciences, Fisheries and Economics

Tromsø, Norway

**Supplementary information**

**Appendix A: Model selection and model outputs**

Table A1. Model selection table results for the mixed-effect model of juvenile growth of Arctic charr against mean water temperature (WT), age of the fish for the back-calculated length increments (1-4 years old), relative abundance shown as Catch-Per-Unit-Effort of the competing part of the charr (<30 cm) and trout (<15 cm) populations for juvenile Arctic charr (CPUEc) and relative abundance shown as Catch-Per-Unit-Effort of the predatory part of the trout population (>25 cm) on juvenile Arctic charr (CPUEp). The table show Aikake`s Information Criterion (AIC), Bayesian Information Criterion (BIC) and the log-likelihood function.

|  | AIC | **BIC** | Log-likelihood |
| --- | --- | --- | --- |
| WT * Age + CPUEc | -255.6 | **-181.6** | 140.8 |
| WT * Age + CPUEc + CPUEp | -260.2 | **-180.5** | 144.1 |
| WT * Age * CPUEc | -236 | **-122.3** | 138.0 |
| WT * Age * CPUEc + CPUEp | -233.0 | **-113.6** | 137.5 |
| WT * Age * CPUEc * CPUEp | -278.9 | **-74.5** | 175.5 |

Table A2. Model selection table results for the mixed-effect model of juvenile growth of Arctic charr against different variables of water temperature measurements (mean autumn temperature (Sep-Nov); MAT, mean growth season temperature (Jun-Nov); MGT, mean annual temperature (Jan-Dec); MAnT, and mean summer temperature (Jun-Aug); MST. The table show Aikake`s Information Criterion (AIC), Bayesian Information Criterion (BIC) and the log-likelihood function.

|  | AIC | **BIC** | Log-likelihood |
| --- | --- | --- | --- |
| MAT | -255.6 | **-181.6** | 140.8 |
| MGT | -200.9 | **-126.9** | 113.5 |
| MAnT | -167.8 | **-93.9** | 96.9 |
| MST | -157.7 | **-83.8** | 91.9 |

Table A3. Scaled linear mixed-effect model results for back-calculated length increment (mm·year^-1^) on a logarithmic scale for 1-4 year old Arctic charr in Lake Takvatn with mean autumn water temperature (MAT) (September-October-November) and relative density of competitors (Arctic charr <30 cm and brown trout <15 cm) (Catch-Per-Unit-Effort-competitors, CPUEc) as fixed effect predictors and fish individuals and study years as random effects.

|  | **Length increment (mm·year^-1^)** | | |
| --- | --- | --- | --- |
| *Predictors* | *Estimates* | *Std.Error* | *P* |
| (Intercept) | 4.215 | 0.0087 | **<0.001** |
| Mean autumn temperature (Sep-Nov) (MAT) | 0.023 | 0.0087 | **0.009** |
| Age 2 years | -0.56 | 0.0122 | **<0.001** |
| Age 3 years | -0.52 | 0.0121 | **<0.001** |
| Age 4 years | -0.54 | 0.0171 | **<0.001** |
| Relative density competition (CPUEc) | -0.011 | 0.005 | **0.047** |
| MTA * Age 2 years | 0.048 | 0.013 | **<0.001** |
| MTA * Age 3 years | 0.024 | 0.013 | 0.056 |
| MTA * Age 4 years | 0.079 | 0.015 | **<0.001** |
| **Random Effects** | | | |
| N _Fish_ | 682 | | |
| N _Years_ | 32 | | |
| Observations | 2195 | | |
| Marginal R^2^ / Conditional R^2^ | 0.566 / 0.595 | | |
|  |  | | |
|  |  | | |
|  |  | | |
| 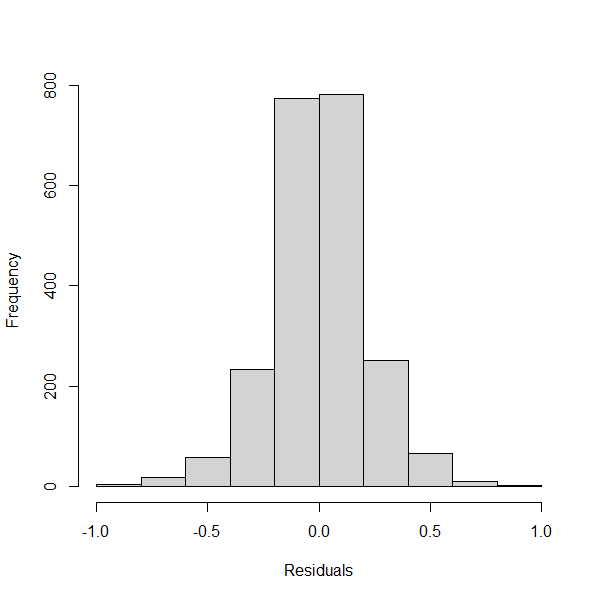 | 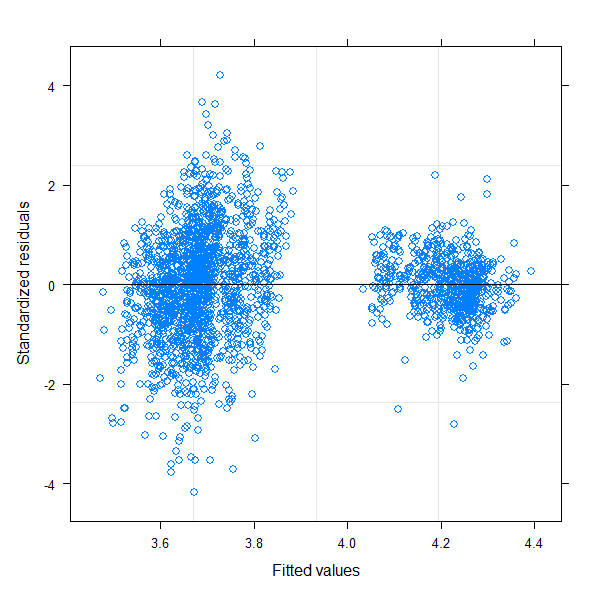 | | |

Figure A1. Left: Histogram showing the distribution of the residuals. Right: Scatterplot of fitted vs. observed variance in the residuals. The plot show two clusters, but the spread of the residuals is consistent across both clusters and there are no outliers raising concern.


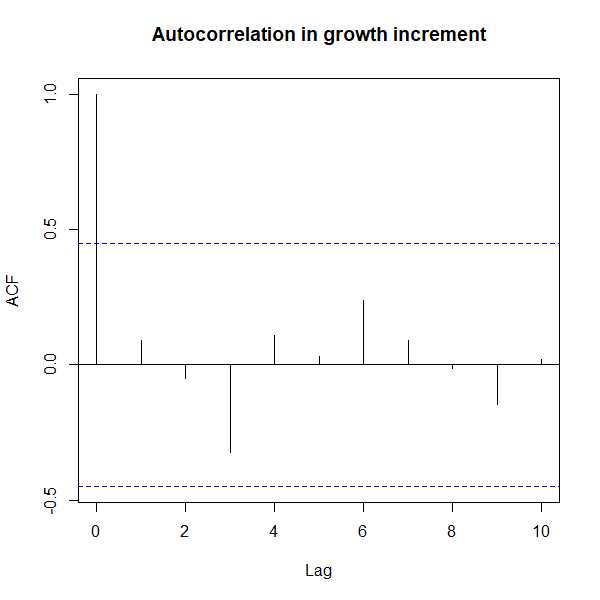

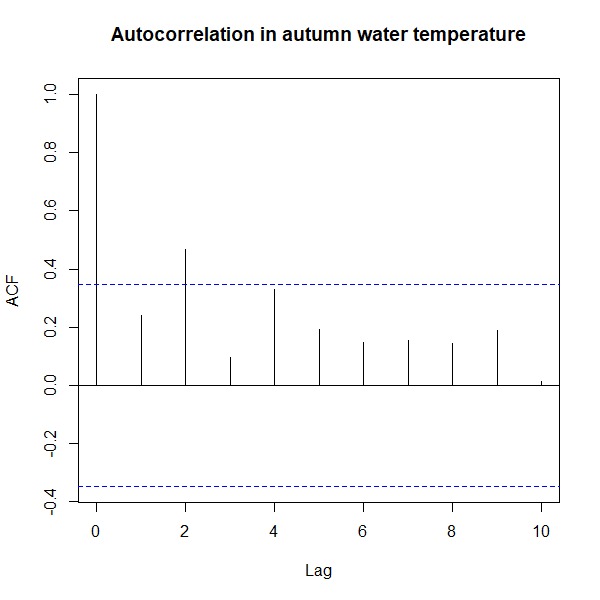

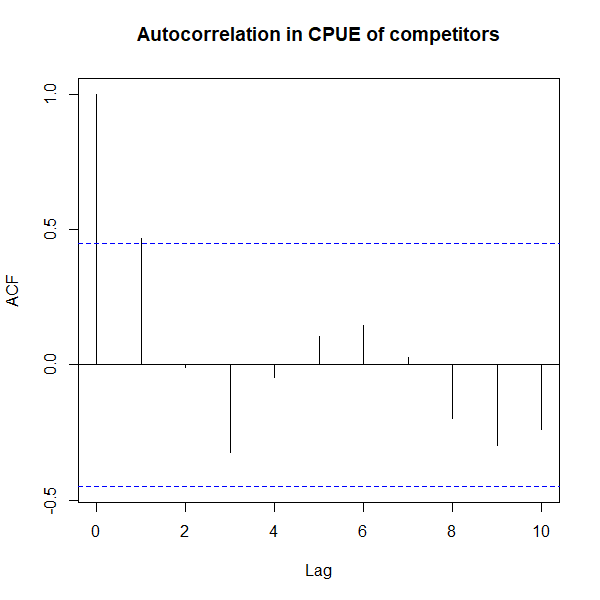


Figure A2. Three panels of autocorrelation plots. Left: Autocorrelation in growth increment estimates. Middle: Autocorrelation in autumn water temperature. Right: Autocorrelation in density (CPUE) of competitors. None of the different variables show any concerning trends regarding autocorrelation.

Table A4. Quantile regression results for back-calculated length increment (mm·year^-1^) dependent on mean autumn water temperature (September-October-November) and relative density of competitors (Arctic charr <30 cm and brown trout <15 cm) (Catch-Per-Unit-Effort-competitors, CPUEc) for 1 year old Arctic charr in Takvatn (50^th^, 75^th^ and 90^th^ percentile).

|  | Percentile | Regression coefficient | Lower 95% CL | Upper 95% CL |
| --- | --- | --- | --- | --- |
| Intercept | 0.5 | 67.97 | 67.40 | 68.56 |
| Water temperature |  | 2.38 | 1.79 | 2.96 |
| CPUEc |  | 0.14 | -0.60 | 0.68 |
| Intercept | 0.75 | 72.95 | 72.2 | 73.7 |
| Water temperature |  | 1.68 | 1.11 | 2.89 |
| CPUEc |  | 0.02 | -0.54 | 0.73 |
| Intercept | 0.90 | 78.1 | 77.13 | 78.88 |
| Water temperature |  | 1.99 | 1.24 | 2.93 |
| CPUEc |  | -0.02 | -1.02 | 0.77 |

Table A5. Quantile regression results for back-calculated length increment (mm·year^-1^) dependent on mean autumn water temperature (September-October-November) and relative density of competitors (Arctic charr <30 cm and brown trout <15 cm) (Catch-Per-Unit-Effort-competitors, CPUEc) for 2 year old Arctic charr in Takvatn (50^th^, 75^th^ and 90^th^ percentile).

|  | Percentile | Regression coefficient | Lower 95% CL | Upper 95% CL |
| --- | --- | --- | --- | --- |
| Intercept | 0.5 | 37.85 | 37.3 | 38.53 |
| Water temperature |  | 2.24 | 1.17 | 3.21 |
| CPUEc |  | -1.16 | -1.66 | -0.48 |
| Intercept | 0.75 | 43.4 | 42.8 | 44.29 |
| Water temperature |  | 2.98 | 1.85 | 3.61 |
| CPUEc |  | -0.21 | -1.06 | 0.21 |
| Intercept | 0.90 | 49.63 | 48.71 | 51.36 |
| Water temperature |  | 4.12 | 2.46 | 6.04 |
| CPUEc |  | -0.77 | -2.17 | 0.79 |

Table A6. Quantile regression results for back-calculated length increment (mm·year^-1^) dependent on mean autumn water temperature (September-October-November) and relative density of competitors (Arctic charr <30 cm and brown trout <15 cm) (Catch-Per-Unit-Effort-competitors, CPUEc) for 3 year old Arctic charr in Takvatn (50^th^, 75^th^ and 90^th^ percentile).

|  | Percentile | Regression coefficient | Lower 95% CL | Upper 95% CL |
| --- | --- | --- | --- | --- |
| Intercept | 0.5 | 41.3 | 40.56 | 42.0 |
| Water temperature |  | 1.88 | 0.92 | 2.85 |
| CPUEc |  | -0.42 | -1.64 | -0.14 |
| Intercept | 0.75 | 49.54 | 48.28 | 50.38 |
| Water temperature |  | 1.62 | 0.37 | 2.73 |
| CPUEc |  | -1.44 | -2.70 | 0.10 |
| Intercept | 0.90 | 57.31 | 56.30 | 59.87 |
| Water temperature |  | 2.01 | -0.28 | 3.70 |
| CPUEc |  | -1.03 | -3.20 | 0.02 |

Table A7. Quantile regression results for back-calculated length increment (mm·year^-1^) dependent on mean autumn water temperature (September-October-November) and relative density of competitors (Arctic charr <30 cm and brown trout <15 cm) (Catch-Per-Unit-Effort-competitors, CPUEc) for 4 year old Arctic charr in Takvatn (50^th^, 75^th^ and 90^th^ percentile).

|  | Percentile | Regression coefficient | Lower 95% CL | Upper 95% CL |
| --- | --- | --- | --- | --- |
| Intercept | 0.5 | 41.92 | 40.42 | 43.28 |
| Water temperature |  | 5.71 | 4.52 | 7.95 |
| CPUEc |  | 0.18 | -0.78 | 2.53 |
| Intercept | 0.75 | 49.98 | 48.78 | 52.14 |
| Water temperature |  | 6.49 | 4.24 | 8.01 |
| CPUEc |  | 2.18 | -1.31 | 3.66 |
| Intercept | 0.90 | 60.03 | 57.43 | 62.13 |
| Water temperature |  | 5.71 | 3.17 | 8.99 |
| CPUEc |  | 0.61 | -1.76 | 6.22 |

**
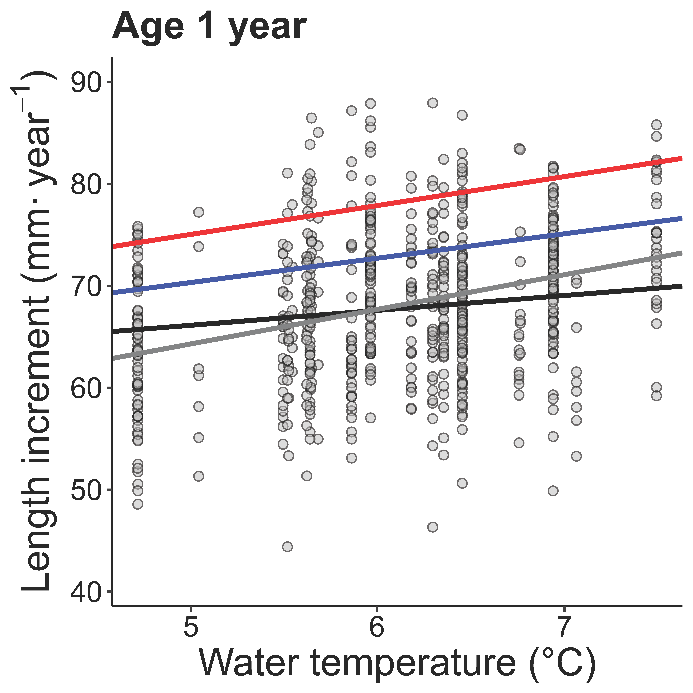

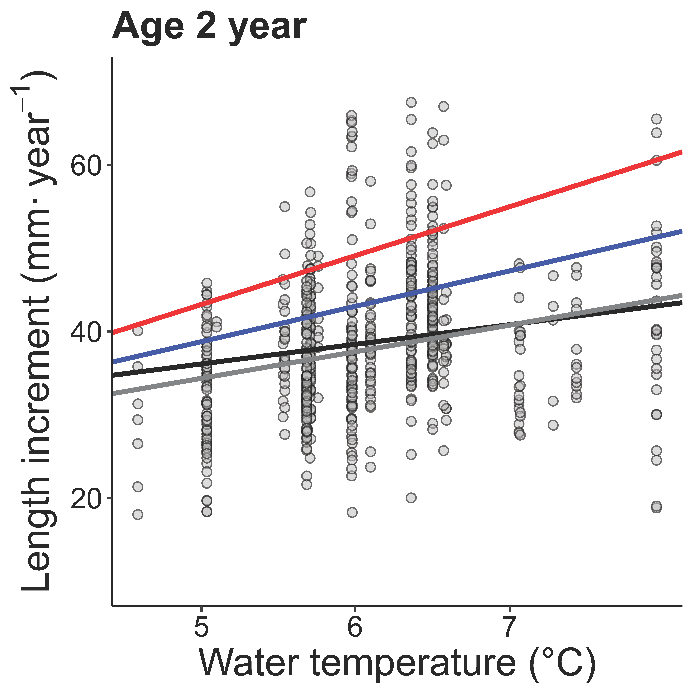
**

**
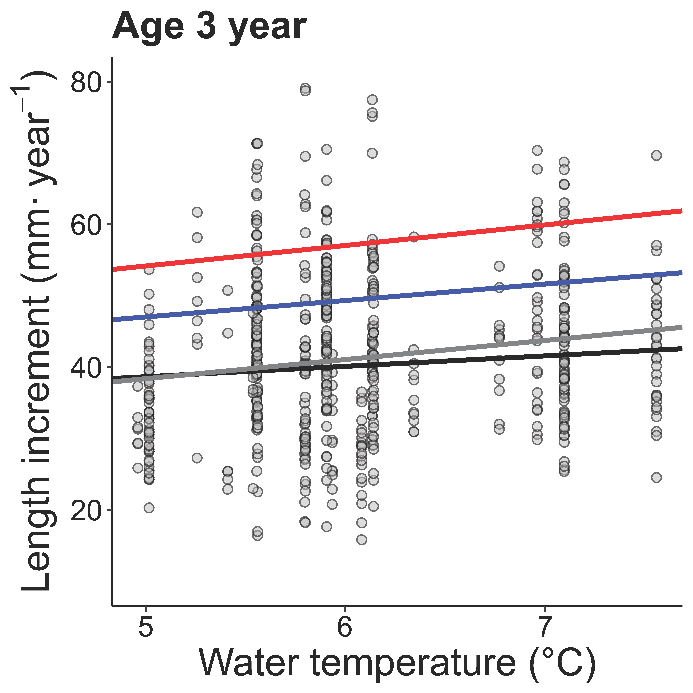

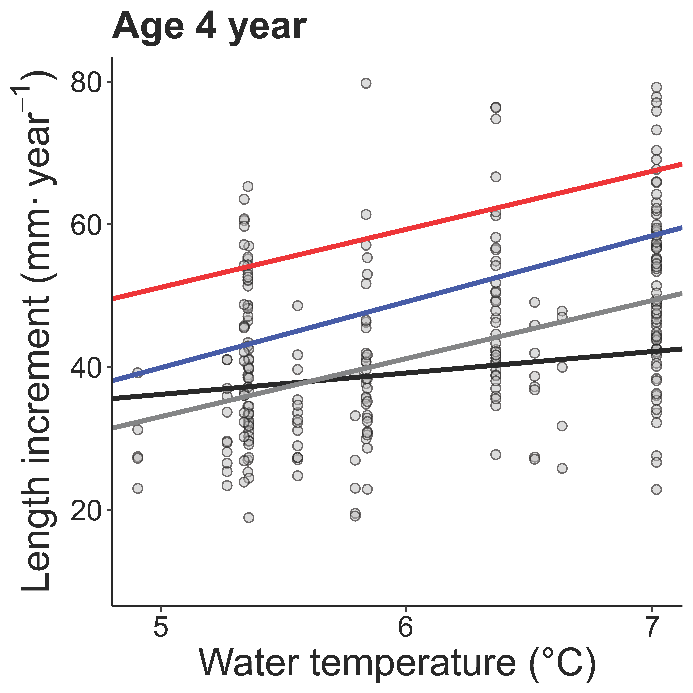
**

Figure A3. Estimated annual somatic length increment (mm · year^-1^) plotted against mean annual autumn water temperature (MAT) (°C) by age group, points show individual fish. The plots for the four age classes include linear and percentile regression lines, with mean (estimated from the mixed-effect model) (black line), median (grey line), 75^th^ (blue line) and 90^th^ (red line) percentiles.

**
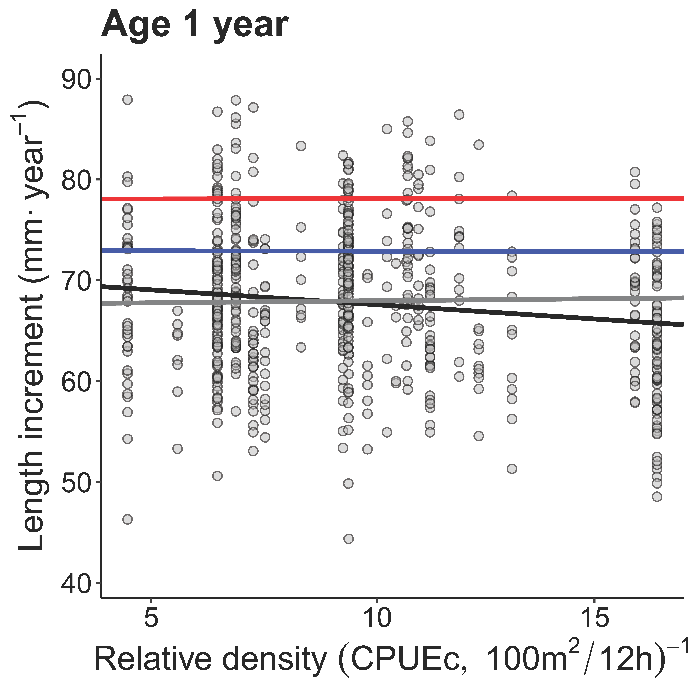

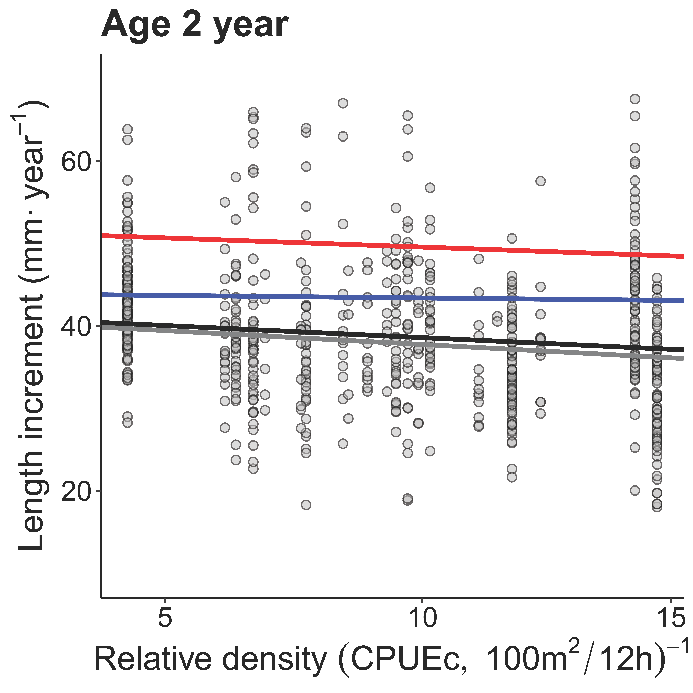
**

**
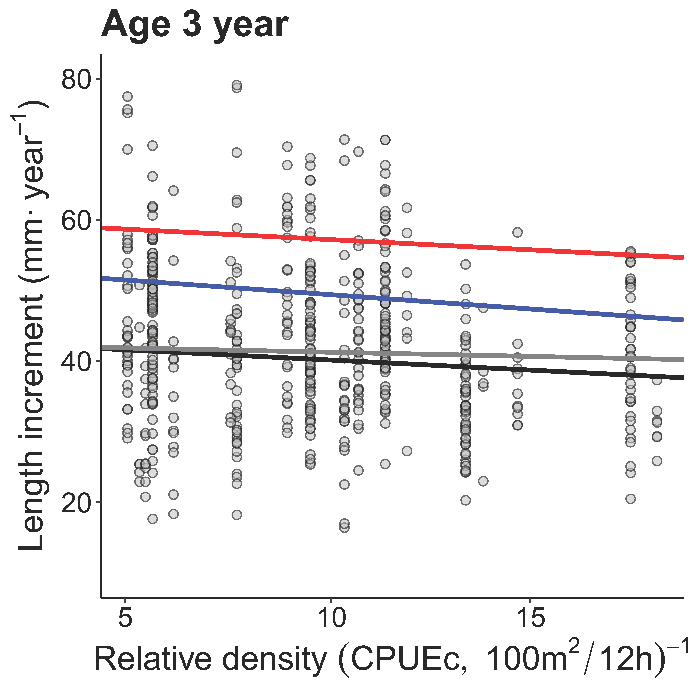

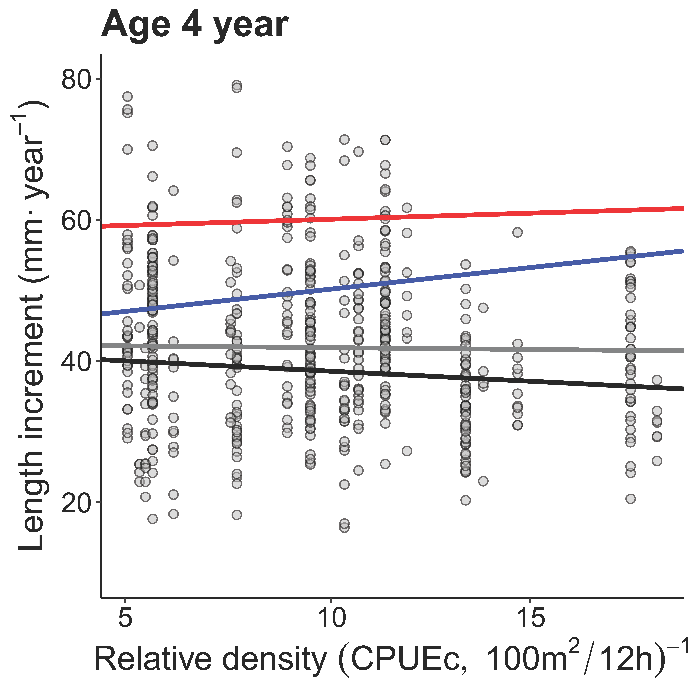
**

Figure A4. Estimated annual somatic growth (mm · year^-1^) plotted against the relative density of competitors (Arctic charr <30 cm and brown trout <15 cm) (Catch-Per-Unit-Effort-competitors, CPUEc) by age group, points show individual fish. The plots for the four age classes include linear and percentile regression lines, with mean (estimated from the mixed-effect model) (black line), median (grey line), 75^th^ (blue line) and 90^th^ (red line) percentiles.

**Appendix B: Water-temperature and GLMr-model evaluation.**


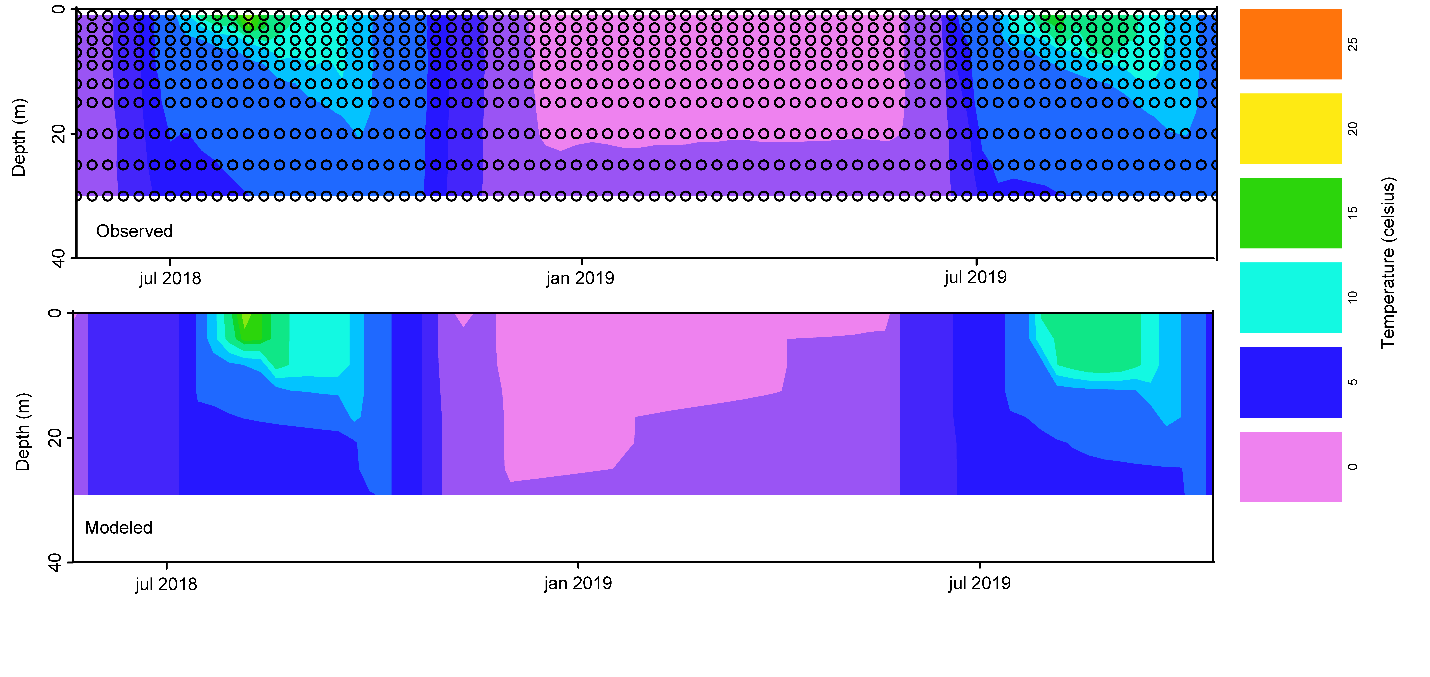
Figure B1. Comparison of observed (top panel) and modelled (bottom panel) water temperature in Lake Takvatn from May 2018 to October 2019. Open circles depict the individual measurements of water temperature by temperature loggers.


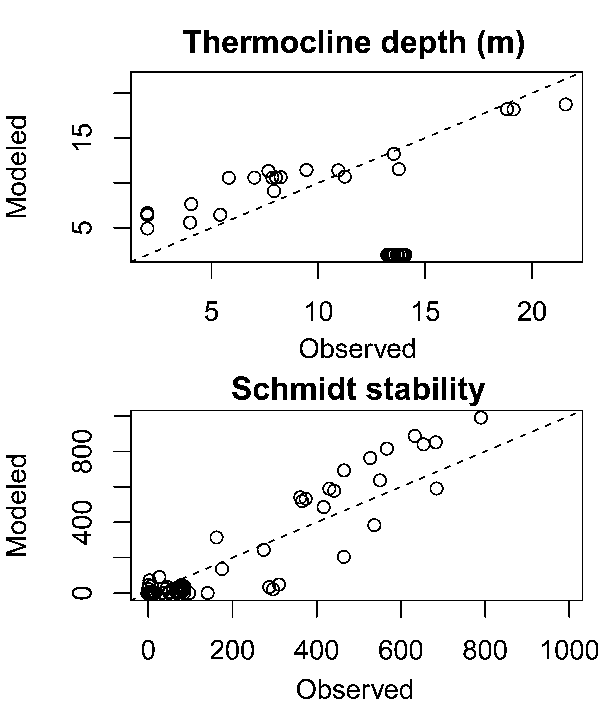

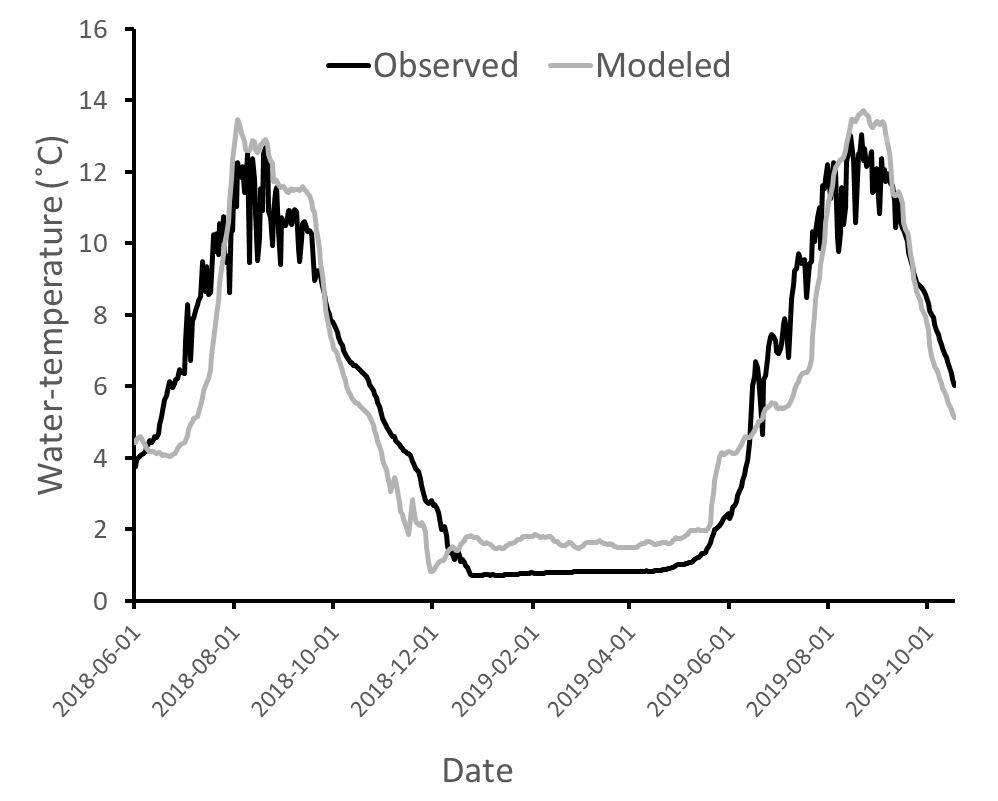
Figure B2. Observed vs. modeled thermocline depth (top left), Schmidt stability (bottom left) and average water temperature (right) for the top ten meters in Lake Takvatn from June 2018 to November 2019.

**
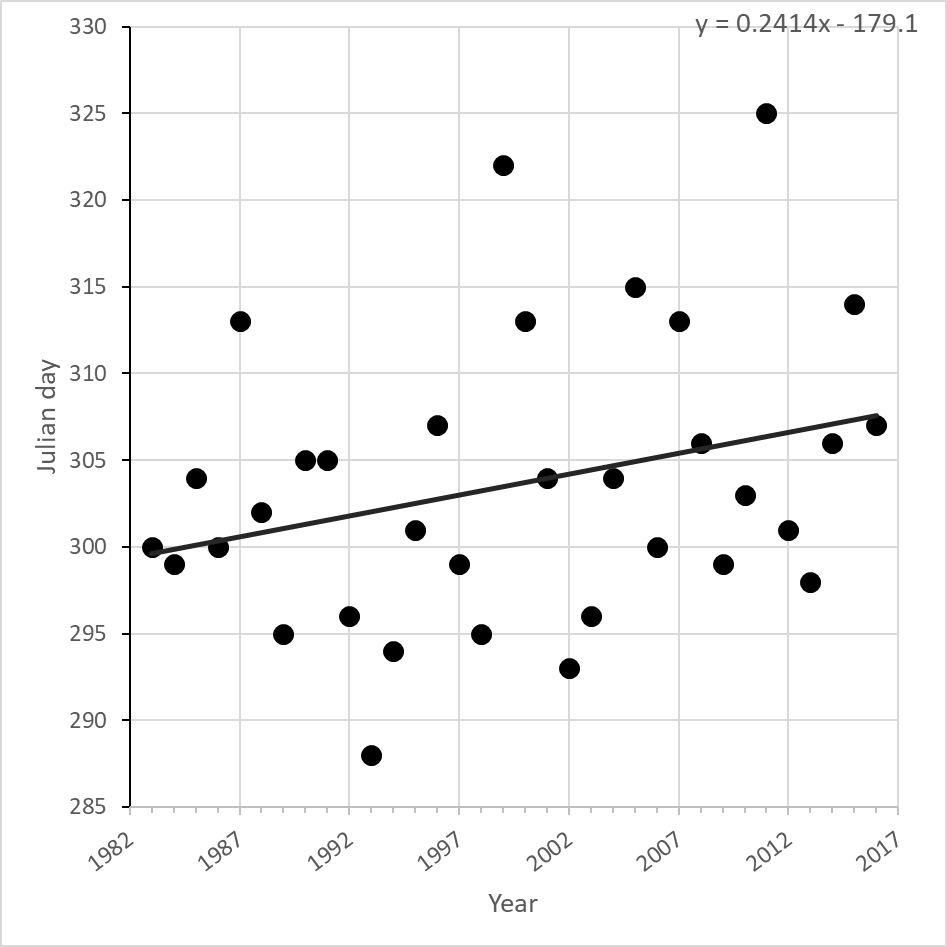

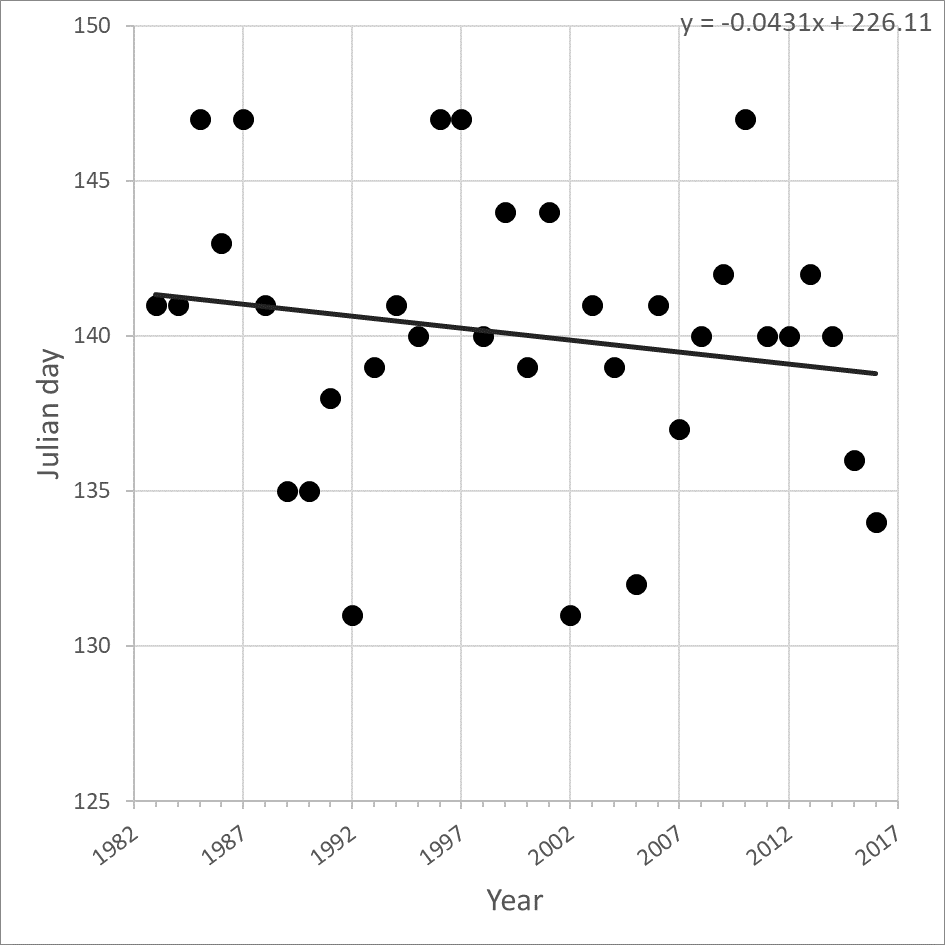
**

Figure B3. Left: Modelled julian day of ice formation in Lake Takvatn from 1983-2016. Right: Modelled Julian day of ice brake-up in Lake Takvatn from 1983-2016.

**Appendix C: Model outputs for Length Increment at age by year throughout the long-term study**

**
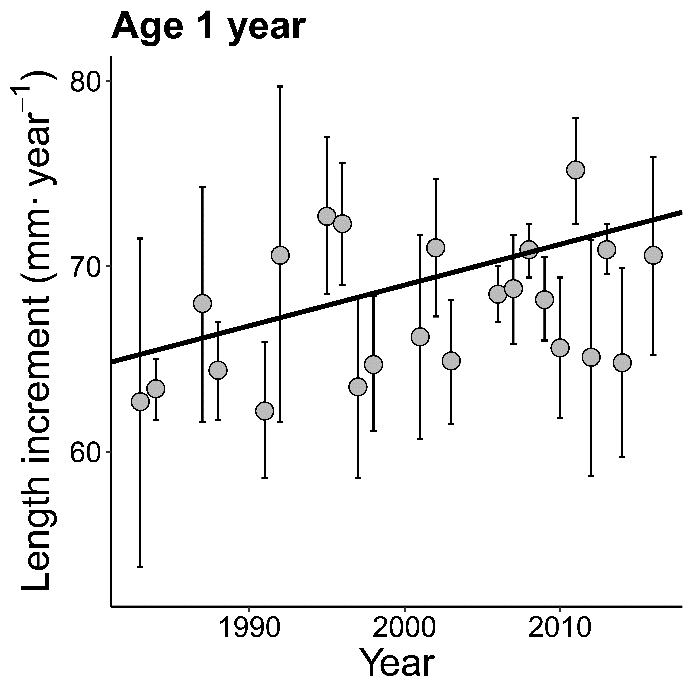

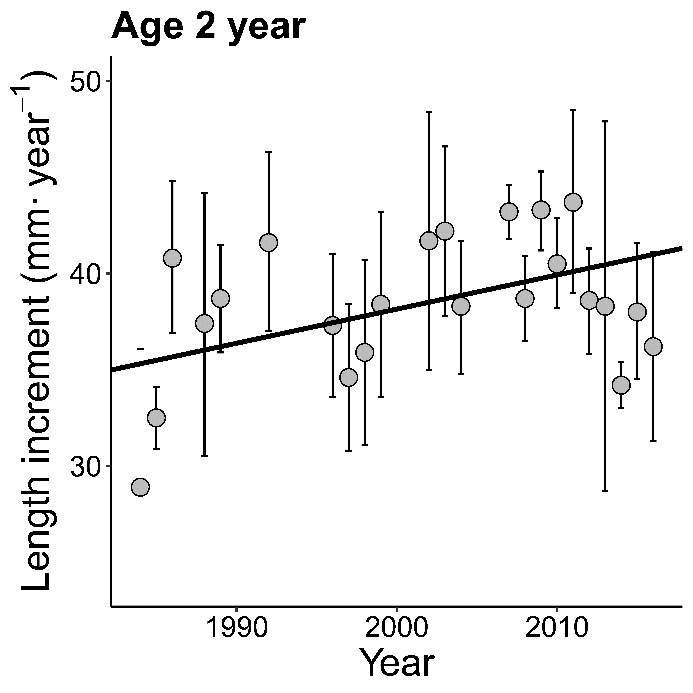
**

**
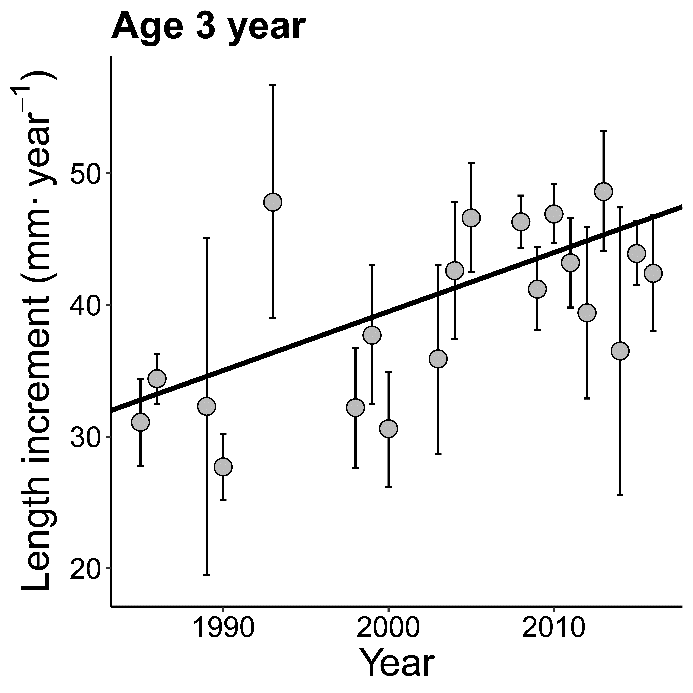

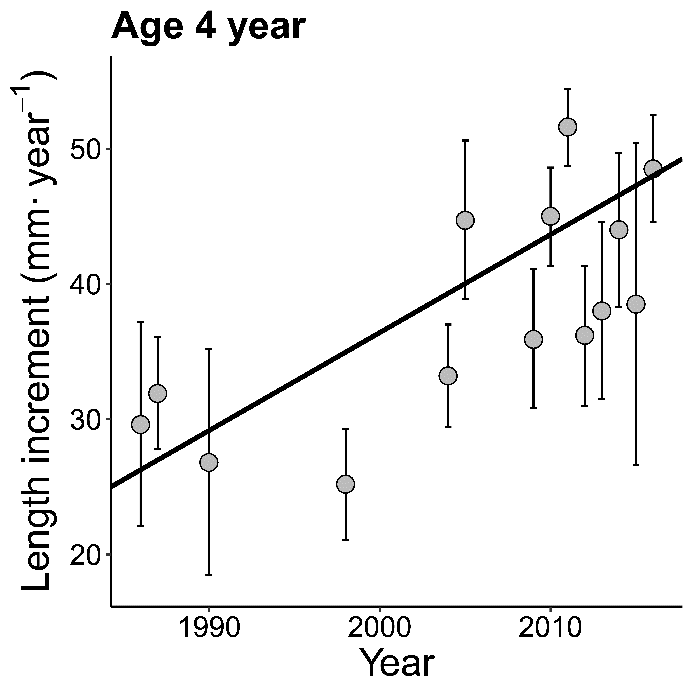
**

Figure C1. Estimated annual length increment (mm · year^-1^) plotted against year by age group. Line represent linear regression and shows the temporal change in growth over the course of the study period.

Table C1. Linear regression model results for back-calculated length increment (mm · year^-1^) with year of the study as predictor for 1 year old Arctic charr in Lake Takvatn.

|  | **Length increment at age 1** | | |
| --- | --- | --- | --- |
| *Predictors* | *Estimates* | *CI* | *p* |
| (Intercept) | 63.95 | 62.41 – 65.48 | **<0.001** |
| Year | 0.22 | 0.15 – 0.28 | **<0.001** |
| Observations | 680 | | |
| R^2^ / R^2^ adjusted | 0.057 / 0.056 | | |

Table C2. Linear regression model results for back-calculated length increment (mm · year^-1^) with year of the study as predictor for 2 year old Arctic charr in Lake Takvatn.

|  | **Length increment at age 2** | | |
| --- | --- | --- | --- |
| *Predictors* | *Estimates* | *CI* | *p* |
| (Intercept) | 35.19 | 33.47 – 36.90 | **<0.001** |
| Year | 0.18 | 0.10 – 0.25 | **<0.001** |
| Observations | 667 | | |
| R^2^ / R^2^ adjusted | 0.032 / 0.031 | | |

Table C3. Linear regression model results for back-calculated length increment (mm · year^-1^) with year of the study as predictor for 3 year old Arctic charr in Lake Takvatn.

|  | **Length increment at age 3** | | |
| --- | --- | --- | --- |
| *Predictors* | *Estimates* | *CI* | *p* |
| (Intercept) | 32.75 | 30.44 – 35.06 | **<0.001** |
| Year | 0.45 | 0.35 – 0.55 | **<0.001** |
| Observations | 620 | | |
| R^2^ / R^2^ adjusted | 0.107 / 0.105 | | |

Table C4. Linear regression model results for back-calculated length increment (mm · year^-1^) with year of the study as predictor for 4 year old Arctic charr in Lake Takvatn.

|  | **Length increment at age 4** | | |
| --- | --- | --- | --- |
| *Predictors* | *Estimates* | *CI* | *p* |
| (Intercept) | 26.28 | 21.63 – 30.92 | **<0.001** |
| Year | 0.73 | 0.54 – 0.93 | **<0.001** |
| Observations | 277 | | |
| R^2^ / R^2^ adjusted | 0.166 / 0.163 | | |

**Appendix D: Summary of the data used in this study.**

Table D1. Summary of data, including sampling year, sample size, mean age and length, and age and length interval of the sampled Arctic charr.

| Sampling year | Sample size (N) | Mean age (years) | Age (min-max) (years) | Mean length (mm) | Length (min-max) (mm) |
| --- | --- | --- | --- | --- | --- |
| 1986 | 19 | 2.1 | 2-3 | 106.9 | 92-137 |
| 1987 | 50 | 3.06 | 2-4 | 132.5 | 104-155 |
| 1988 | 11 | 3.9 | 3-4 | 160.4 | 128-194 |
| 1991 | 29 | 3.24 | 2-5 | 132.6 | 84-202 |
| 1995 | 15 | 3.53 | 3-4 | 205.4 | 137-241 |
| 1999 | 26 | 3.5 | 3-4 | 145.9 | 114-209 |
| 2000 | 11 | 3.1 | 3-4 | 132.4 | 114-198 |
| 2001 | 12 | 3.02 | 3-4 | 130 | 106-160 |
| 2005 | 42 | 3.31 | 3-4 | 152.9 | 103-275 |
| 2006 | 56 | 3.36 | 3-4 | 147.7 | 86-250 |
| 2009 | 76 | 3.01 | 2-4 | 145.2 | 110-185 |
| 2010 | 18 | 3.44 | 3-4 | 163.2 | 122-204 |
| 2012 | 98 | 4.11 | 2-7 | 209.2 | 93-333 |
| 2013 | 68 | 4.34 | 3-7 | 206.2 | 99-354 |
| 2014 | 18 | 3.56 | 3-5 | 156.4 | 113-197 |
| 2015 | 20 | 3.95 | 3-4 | 186.1 | 138-243 |
| 2016 | 47 | 3.04 | 3-4 | 142.6 | 105-184 |
| 2017 | 52 | 3.85 | 3-5 | 165.2 | 105-222 |
| 2018 | 17 | 2.53 | 2-3 | 113.1 | 95-146 |
| = | 685 | 3.49 | 2-7 | 162.2 | 84-333 |
